# *Exophiala dermatitidis* isolates from various sources: using alternative invertebrate host organisms (*Caenorhabditis elegans* and *Galleria mellonella*) to determine virulence

**DOI:** 10.1038/s41598-018-30909-5

**Published:** 2018-08-24

**Authors:** Maike Olsowski, Frederike Hoffmann, Andrea Hain, Lisa Kirchhoff, Dirk Theegarten, Daniel Todt, Eike Steinmann, Jan Buer, Peter-Michael Rath, Joerg Steinmann

**Affiliations:** 1 Institute of Medical Microbiology, University Hospital Essen, University of Duisburg-Essen, Essen, Germany; 2 Institue of Pathology, University Hospital Essen, University of Duisburg-Essen, Essen, Germany; 3 Institute for Experimental Virology, TWINCORE Centre for Experimental and Clinical Infection Research; a joint venture between the Medical School Hannover (MHH) and the Helmholtz Centre for Infection Research (HZI), Hannover, Germany; 4 Department of Molecular and Medical Virology, Ruhr-University Bochum, Bochum, Germany; 5 Institute of Clinical Hygiene, Medical Microbiology and Infectiology, Klinikum Nürnberg, Paracelsus Medical University, Nuremberg, Germany

## Abstract

*Exophiala dermatitidis* causes chromoblastomycosis, phaeohyphomycosis and fatal infections of the central nervous system of patients with Asian background. It is also found in respiratory secretions from cystic fibrosis (CF) patients. In this study a variety of *E. dermatitidis* strains (isolates from Asia, environmental and CF) were characterized in their pathogenicity by survival analyzes using two different invertebrate host organisms, *Caenorhabditis elegans* and *Galleria mellonella*. Furthermore, the morphological development of hyphal formation was analyzed. *E. dermatitidis* exhibited pathogenicity in *C. elegans*. The virulence varied in a strain-dependent manner, but the nematodes were a limited model to study hyphal formation. Analysis of a melanin-deficient mutant (Mel-3) indicates that melanin plays a role during virulence processes in *C. elegans*. The strains isolated from Asian patients exhibited significantly higher virulence in *G. mellonella* compared to strains from other sources. Histological analyzes also revealed a higher potential of invasive hyphal growth in strains isolated from Asian patients. Interestingly, no significant difference was found in virulence between the Mel-3 mutant and their wild type counterpart during infection in *G. mellonella*. In conclusion, invasive hyphal formation of *E. dermatitidis* was associated with increased virulence. This work is the basis for future studies concerning *E. dermatitidis* virulence.

## Introduction

The black yeast-like fungi are a group of species defined by morphological aspects: sexual dimorphism, no known sexual reproduction, and a black-brownish color caused by the production of 1,8-dihydroxynaphthalene (DHN) melanin. Because of their unknown teleomorphic life cycle, these fungi cannot be classified in the established system of fungal taxonomy^1^. All members of the genus *Exophiala* are classified as black yeast-like fungi^1,2^. However, genetic analysis of this genus classifies *Exophiala* into the phylum of Ascomycota^1,3^. The polymorphic property of *Exophiala dermatitidis* (Kano) de Hoog, also known as *Wangiella dermatitidis* (Kano) McGinnis, allows mitotic reproduction in a yeast-like manner by the production of budding cells (yeast phase), in chains of budding cells called pseudohyphae, or in a hyphal formation by creating true septate hyphae^1,4^. Hyphal development begins with a single fungal cell, which produces pseudohyphal structures before developing true hyphae^1,4,5^. The described yeast-hyphal transition is considered to be a virulence factor of dimorphic fungi, as has been described for *Candida albicans*^4,6,7^, and has also been previously discussed as a virulence factor for *E. dermatitidis*^8^.

*E. dermatitidis* is oligotrophic and thermophilic, with a temperature tolerance ranging from 4 °C to 42 °C^1,4^. It has been postulated that this fungus originated in the environment of the rain forest. In Europe it can be found in dishwashers, sauna facilities, and other locations with similar conditions (warm and wet); thus, it has found a new ecological niche outside the tropical climatic zones^9–11^. The clinical relevance of *E. dermatitidis* is reflected in reports of invasive infections after trauma and of phaeohyphomycosis, especially among immune-deficient patients^11^. Fatal infections involving the central nervous system (CNS) have been reported in South and East Asia. Interestingly, all of these patients were reported to be immunocompetent^12,13^. The reason for the higher incidence of *E. dermatitidis* CNS infections among Asian patients is still unexplained^10^. On the other hand, *E*. *dermatitidis* has been recovered from 5% to 19% of respiratory samples from patients with cystic fibrosis (CF)^14,15^. Recently, *E. dermatitidis* has been shown to contribute to inflammation and worsening lung function among CF patients^16^.

In the study reported here, we analyzed *E. dermatitidis* strains from various sources to determine their virulence as related to their origin of isolation. To do so, we created three groups of *E. dermatitidis* strains: one group of environmental origin from Europe and Asia (E); one group of clinical origin isolated from Asian patients with mainly systemic infections (P); and one group of clinical origin isolated from CF patients (CF). To analyze the virulence of the *E. dermatitidis* strains, we used the invertebrate species *Caenorhabditis elegans* and *Galleria mellonella* as infection models.

## Results

### *E. dermatitidis* exhibits pathogenicity in *C. elegans*

*E. dermatitidis* cultures in yeast phase were used for oral infection of *C. elegans*, as has been previously described for *Cryptococcus neoformans*^17^. Nematodes that were fed with *Escherichia coli* OP50 did not die during the seven days of life-span measurement. On the other hand, 70% of the nematodes fed with *C. neoformans* died within 7 days, a finding in line with those of previous studies^17^.

**Figure 1:**
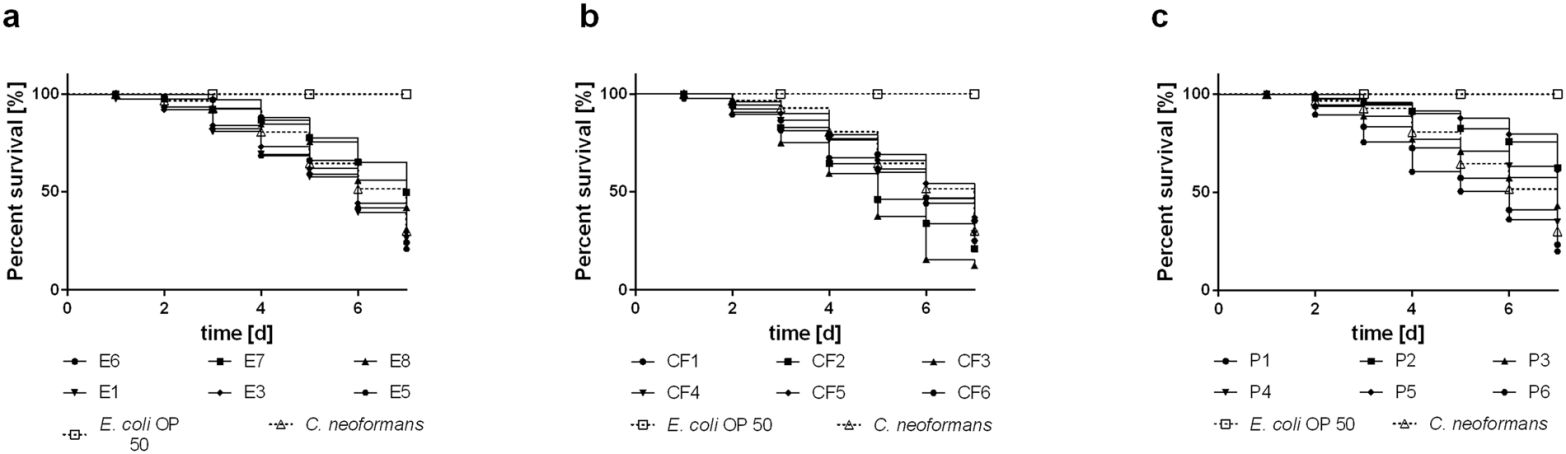
Survival of *C. elegans* after infection with *E. dermatitidis* strains from various sources. (**a**) Isolates from the environment (E); (**b**) Isolates from cystic fibrosis patients (CF); (**c**) Isolates from Asian patients (P). *C. neoformans* was used as an infection control; *E. coli* OP 50 was used as a non-infection control. *E. dermatitidis* exhibited pathogenicity in the *C. elegans* assay. Mortality rates varied between fungal strains. n = 30–40 individuals per test were used, every test was repeated 3 times.

*C. elegans* has been previously used as a model host organism for analyzing the pathogenesis and virulence factors of a plurality of bacteria, such as *Staphylococcus aureus* and *Pseudomonas aeruginosa*^18^. However, the pathogenic properties of only a few fungal species, including *C. neoformans* and *C. albicans*, have been determined by using *C. elegans*^17–19^. Because the nematode’s body is transparent, the activity of the microorganism during infection with *C. elegans* can be easily observed. The clinical picture of microorganisms in *C. elegans* depends primarily on their developed virulence factors, which are also involved in mammalian-pathogen interactions during infection^18,20^. To determine whether the source specificity of various strains influences virulence or pathogenicity, we infected *C. elegans* with 18 strains of *E. dermatitidi*s originating from the three separate sources of origin: E (strain E1, E3, and E5–8), P (strains P1–6), and CF (strains CF1–6). All of the tested *E. dermatitidi*s strains were able to kill *C. elegans* (Fig. 1a–c). The life span of nematodes feeding on *E. dermatitidis* strains tended to be strain-dependent: the portion of infected *C. elegans* populations that survived for seven days ranged from 12% to 60%. The comparison of mean-survival and median-survival rates for the three strain groups indicates a parametric distribution of virulence behaviour within each strain group (Table 1). However, when we compared the group-specific survival curves with a log-rank test, we found no differences between the tested strain groups in the killing of *C. elegans* by *E. dermatitidis* (comparison of CF with E, *P* = 0.41; comparison of E with P, *P* = 0.39; comparison of P with CF, *P* = 0.10). For more information about the used strains and strain groups see also Table 2.

**Table 1:** Identification of parametric distribution of data by comparison of means and medians of median survival.

	Strain group	Mean	Median	
*C. elegans*	E	6.3	6.0	Parametric
median-	CF	5.83	6.0	Parametric
survival	P	7.0	7.0	Parametric
*G. mellonella*	E	6.5	7.0	Parametric
median-	CF	6.9	7.25	Parametric
survival	P	4.75	4.75	Parametric

A difference of 1.0 or lower indicated parametric data. Median-survival rates of P2 and P5 were determined by calculating the intercept as y = 50%.

**Table 2:** Source and origin of included *Exophiala dermatitidis* strains.

*	Species	Strain	Source	Origin
E1	*Exophiala dermatitidis*	CBS 120550	Steam bath	Austria
E3	*Exophiala dermatitidis*	CBS 109142	Beer surface	Netherland
E5	*Exophiala dermatitidis*	CBS 120479	Air	Germany
E6	*Exophiala dermatitidis*	CBS 106.92	Public bath	Japan
E7	*Exophiala dermatitidis*	CBS 120435	Steam bath	Thailand
E8	*Exophiala dermatitidis*	CBS 120574	Sauna facility	Thailand
P1	*Exophiala dermatitidis*	CBS 109154	Brain	Korea
P2	*Exophiala dermatitidis*	CBS 116372	Brain	Japan
P3	*Exophiala dermatitidis*	CBS 123467	Liquor	Japan
P4	*Exophiala dermatitidis*	CBS 578.76	Brain, chromomycosis	Taiwan
P5	*Exophiala dermatitidis*	CBS 577.76	Brain, cervical lymph node	Taiwan
P6	*Exophiala dermatitidis*	CBS 579.76	Brain	Japan
CF1	*Exophiala dermatitidis*	CBS 154.90	Sputum from CF patient^†^	Germany
CF2	*Exophiala dermatitidis*	CBS 552.90	Sputum from CF patient^†^	Germany
CF3	*Exophiala dermatitidis*	CBS 149.90	Sputum from CF patient^†^	Germany
CF4	*Exophiala dermatitidis*	CBS 120429	(Unknown) from CF patient	Finland
CF5	*Exophiala dermatitidis*	1951	Sputum from CF patient^‡^	Germany
CF6	*Exophiala dermatitidis*	1946	Sputum from CF patient^‡^	Germany
Mel^−3^	*Exophiala dermatitidis*	ATCC 44504	Melanin-deficient mutant	‡
WT	*Exophiala dermatitidis*	ATCC 34100	Wild-type strain of Mel^−3^	‡
	*Cryptococcus neoformans*	CBS 464	Reference stock	†
	*Candida albicans*	ATCC 90028	Reference stock	†
	*Escherichia coli* OP50			^¶^
	*Caenorhabditis elegans* N2			‡
	*Galleria mellonella*		Zoo und Angelsport GmbH	Germany

*Identification nomenclature used in this study: E, environmental strains; P, strains from Asian patients; CF, strains isolated from patients with cystic fibrosis. ^†^Institute of Medical Microbiology, Essen, Germany; ^‡^Institute of Medical Microbiology, Aachen, Germany; ^¶^Institute of Medical Microbiology, Bochum, Germany.

### The presence of infection-related hyphal structures after proliferation into *C. elegans* is isolation source-dependent

**Figure 2:**
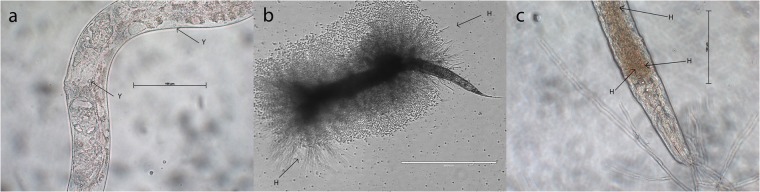
Morphological analysis of *E. dermatitidis* in *C. elegans* after infection and incubation of infected nematodes for 72 h. Three separate pictures of *E. dermatitidis* development in *C. elegans* could be observed, leading to death: (**a**) Adherence of mainly fungal budding cell on the intestinal wall; proliferation and obstipation continued until no free intestinal structure could be observed, leading to death. (**b**,**c**) Invasive hyphal structures from inside the intestine to outside, leading to death. Scale = 400 µm.

We determined the development of fungal morphologic structures in infected *C. elegans* nematodes. These were inoculated over a period of 24 h and 48 h by oral inoculation, isolated from fungi cultures, and were incubated for 72 h. We could observe morphologic structures such as budding cells, pseudohyphae-like cell structures, and septate hyphal structures. We selected the following two characteristic features of *E. dermatitidis* development during infection with *C. elegans*: (A) yeast-phase proliferation in the intestine of *C. elegans*, no visible hyphal structures, death caused by obstipation and bursting (Fig. 2a,b) Hyphal formation (noninvasive and invasive hyphae; Fig. 2b,c).

**Figure 3:**
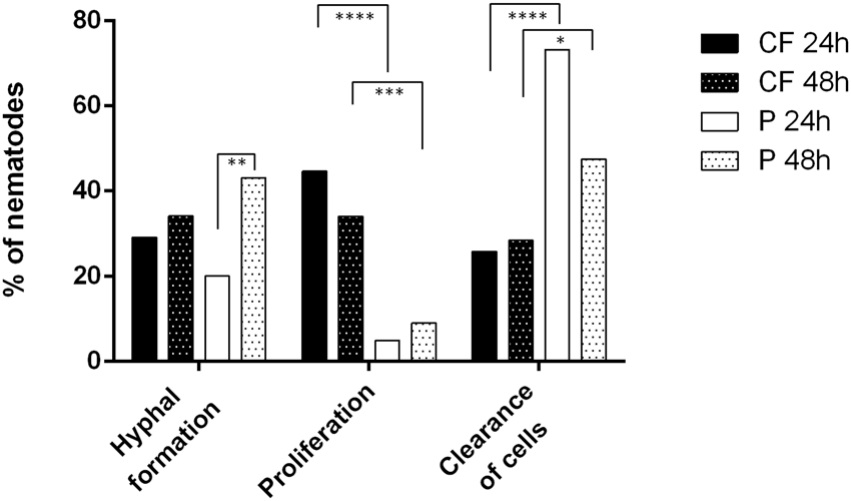
Statistical evaluation of the morphology of *E. dermatitidis* in *C. elegans* during infection. Strains from cystic fibrosis patients (CF) strains exhibited no significant differences in morphological development after infection for 24 h or 48 h. Strains from Asian patients (P) exhibited significantly more hyphal formation after infection for 48 h than after infection for 24 h. Rates of nematodes were calculated with Kaplan-Meier statistics; comparison were performed with Mantel-Cox log-rank tests with statistical significance set at the level of *P* ≤ 0.05. *P ≤ 0.05; **P ≤ 0.01; ***P ≤ 0.001; ****P ≤ 0.0001, n = 24.

After 24-h inoculation and three days of incubation, yeast-phase proliferation in the intestine, leading to obstipation and death by bursting of the cuticula (40%), and hyphal structures (29%) were observed more often in CF strains than in P strains (Fig. 3). Nematodes infected with P-strains showed hyphal structures only in a rate of 20%, not more than 9% showed proliferation with obstipation in the intestine. More than 70% of nematodes inoculated with P strains were able to remove fungal cells from the intestine. However, after an incubation of 48 h, 47% of nematodes infected with P strains exhibited a statistically significant increase in the incidence of infection-related hyphal structures (43%; *P* = 0.0036) combined with a significant decrease in the ability of nematodes to remove fungal cells (47%; *P* = 0.0009). In contrast, CF strains exhibited no statistically significant differences after being inoculated over a time of 24 h or 48 h.

### *E. dermatitidis* shows pathogenicity in *G. mellonella* in an isolation source-dependent manner

The caterpillar larva of the great wax moth *G. mellonella* is also commonly used as a model host organism for infection experiments aimed at analyzing the virulence of microorganism^21^. Compared to *C. elegans* nematodes, the caterpillar insects are formally more sophisticated in their anatomy, development, and immune system. Although the immune response of *C. elegans* is limited in physical options, such as the cuticular barrier and various chemical compounds,^22,23^ the immune system of *G. mellonella* also includes a humoral immune response and specific immune cells, located in the haemolymph^24^.

During the experiments, all infected caterpillars exhibited the typical shift in coloration from yellow to gray within the first 30 min after infection, caused by their own immune response. The control caterpillars treated with phosphate-buffered saline (PBS) exhibited no differences in coloration after injection. Because of these differences between infected and non-infected caterpillars, the finding of no change in coloration within the first 30 min served as an indicator of successful infection.

**Figure 4:**
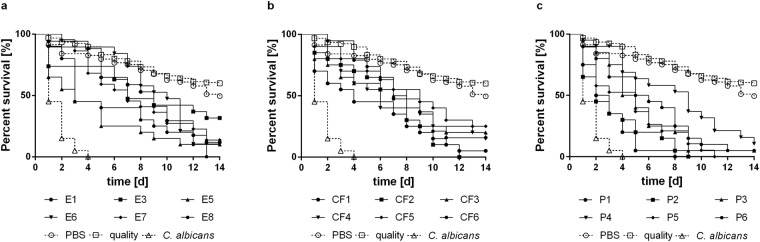
Survival of *G. mellonella* after infection with wild-type strains of *E. dermatitidis* from various sources: **(a**) Isolates from the environment (E). (**b**) Isolates from cystic fibrosis patients (CF). (**c**) Isolates from systemic infections in patients from Asia (P). C. albicans was used as an infection control, sterile phosphate-buffered saline (PBS) was injected as a non-infection control, and a group of unharmed caterpillars were used as a quality control of the caterpillar population. All tested *E. dermatitidis* strains exhibited pathogenicity against *G. mellonella* caterpillars. n = 10 individuals per test were used, every test was repeated 3 times.

**Figure 5:**
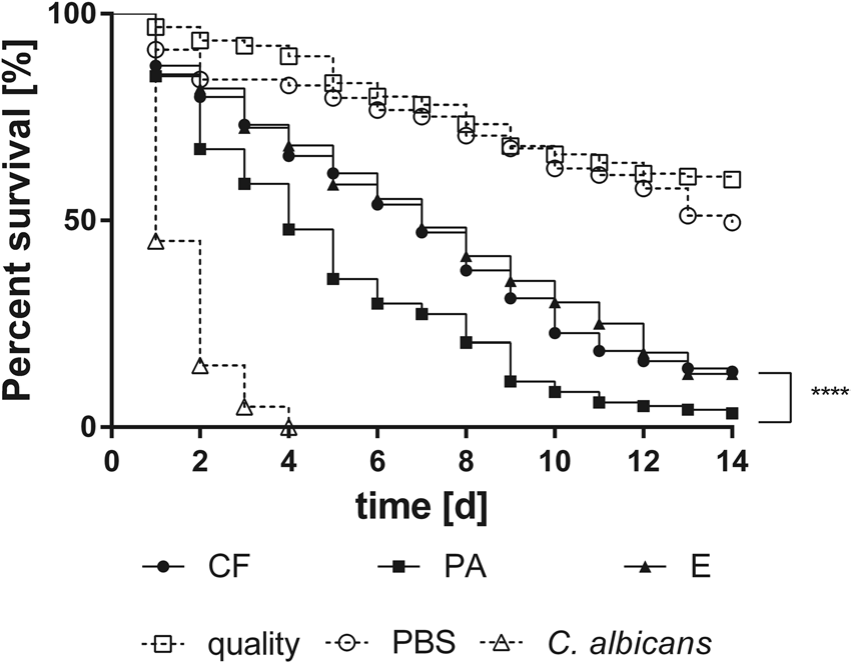
Comparison of the mean survival curves of the three strain groups of *E. dermatitidis* isolates: isolates from cystic fibrosis patients (CF), isolates from Asian patients with systemic infections (P), and environmental isolates (E). P strains exhibited a significantly higher virulence against *G. mellonella* than E strains or CF strains. Curves were compared with the Mantel-Cox log-rank test with significance set at the level of *P* ≤ 0.05. *P ≤ 0.05; **P ≤ 0.01; ***P ≤ 0.001; ****P ≤ 0.0001.

All tested *E dermatitidis* strains showed pathogenicity against *G. mellonella* caterpillars in a strain-dependent manner (Fig. 4). However, comparison of the median-survival of each of the three strain groups indicates that P strains are more virulent than CF or E strains, and comparison of means and medians of the median-survival indicates that the data within strain groups are parametric (Table 1). These parametric data allowed the evaluation of mean survival curves for all three strain groups. P strains were significantly more virulent than the E and CF strains (Fig. 5; comparison of CF and E, *P* = 0.6940; comparison of E and P, *P* < 0.0001; comparison of P and CF, *P* < 0.0001).

### *E. dermatitidis* is able to proliferate into *G. mellonella*

**Figure 6:**
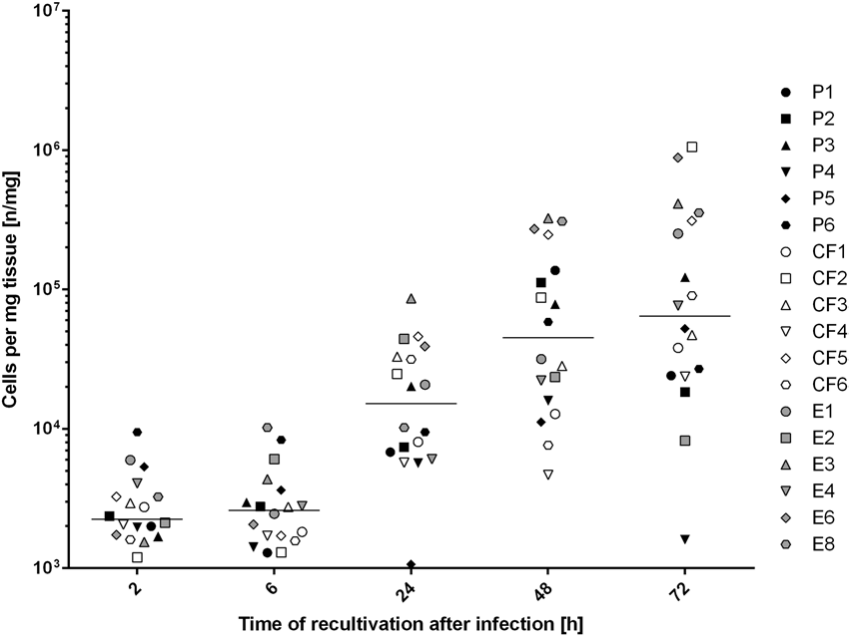
Kinetics of fungal proliferation in *G. mellonella* during infection. *E. dermatitidis* cells were recovered from infected *G. mellonella* caterpillars 2 h, 6 h, 24 h, 48 h, and 72 h after infection by CFU experiment (n = 18 strains, 6 from each group). Growth curve shows a sigmoidal development, all tested strains showed a similar growth rate over a period of 72 h. n = 2 *G. mellonella* caterpillars per test were used, every test was repeated 3 times.

**Figure 7:**
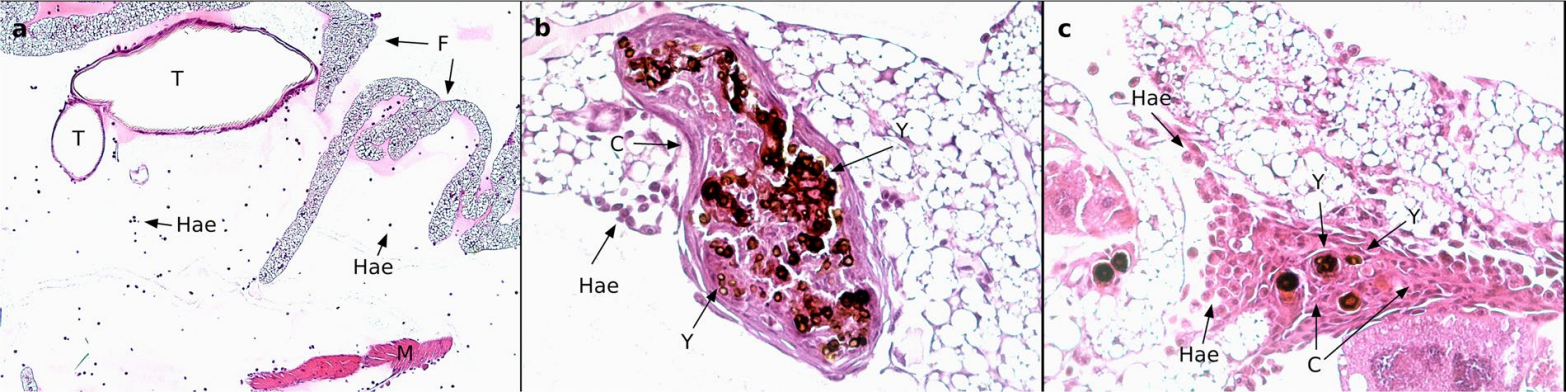
Histopathologic evaluation of *E. dermatitidis* infection in *G. mellonella*. (**a**) No sign of immune system activity in the PBS control 72 h after infection. (**b**) Immune activity 48 h after infection. Haemocytes are creating massive capsule structures around fungal cells. Fungal cells of *E. dermatitidis* are still alive and the budding process is continuing. (**c**) Immune response against fungal cells also continues 72 h after infection. Slides were stained with hematoxylin and eosin (H&E) and were observed with a light microscope at a magnification of 400x (Hae = Haemocytes, T = Trachea, M = Muscle, F = Fett-Body, C = Capsule, Y = Yeast).

**Figure 8:**
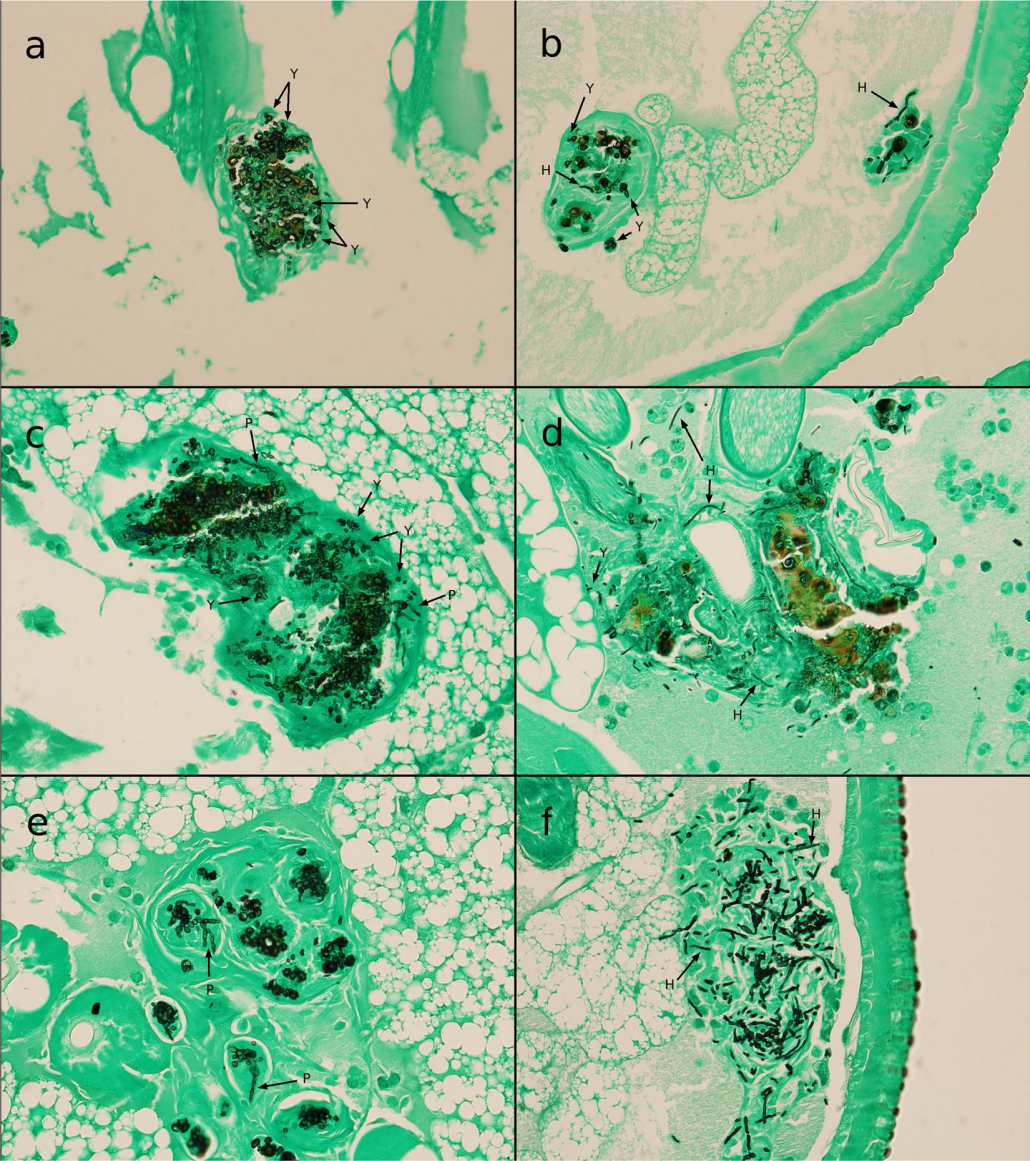
Histopathological analysis of *E. dermatitidis* in *G. mellonella* and comparison between a CF strain and a P strain infection. Shown is a typical strain from a cystic fibrosis (CF) patient with no real hyphal structures within the first 72 h after infection; pseudohyphal structures begin to develop within 48 h (**a**) 24 h after infection; (**c**) 48 h after infection; (**e**) 72 h after infection). A strain from an Asian patient (P) with a systemic infection shows the growth of true hyphal structures within 24 h after infection and a massive invasion of hyphae over a period of 72 h (**b**. 24 h after infection; (**d)** 48 h after infection; (**f**) 72 h after infection). All strains were observed with a light microscope at a magnification of 400x. Slides for histologic analysis were stained with Grocott silver. (H = Hyphae, P = Pseudohyphae, Y = Yeast).

Evaluation of the development of *E. dermatitidis* during infection in *G. mellonella* showed that proliferation of the fungi could be observed from 2 h to 72 h after infection in all tested strains, and in a sigmoidal shape (Fig. 6). We examined prepared and stained slides of tissue from infected and uninfected *G. mellonella* caterpillars 24 h, 48 h, or 72 h after infection. The slides obtained from uninfected PBS control caterpillars, stained with haematoxylin and eosin (H&E) for contrast, showed all of the typical anatomical structures of such an organism. Hemocytes could be observed in the hemolymph, showing a smooth arrangement over the entire area of the slide. In contrast, hemocytes on tissue slides obtained from infected caterpillars (Fig. 7) exhibited behavior typical of that associated with infection: they were concentrated at the area of fungal cells and created massive capsular structures around the pathogenic organisms. Capsular structures exhibited massive growth during the observed infection time of 72 h. Additional contrast was obtained by staining the fungi with Grocott silver (Fig. 8). Observation of all infected strains showed the typical morphologic structures of budding cells in yeast phase, pseudohyphae, and true hyphae. Histologic analysis showed that 3 of 6 P-strain infections exhibited invasive hyphal structures within the first 24 h after infection. One of 6 E strains and all CF strains exhibited hyphae 48 h after infection, and 5 P strains exhibited invasive hyphal structures. At 72 h after infection, true hyphal structures were found in only 2 CF strains, whereas hyphal growth had proliferated in P strains and had begun to invade the tracheal organs of the caterpillar.

### Melanin contributes to the virulence of *E. dermatitidis* as detected in a *C. elegans* infection model, but not in *G. mellonella*

**Figure 9:**
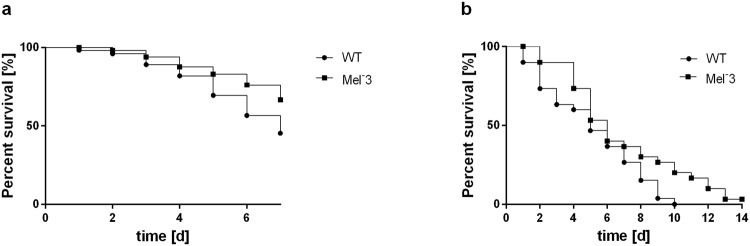
Analysis of survival of *G. mellonella* and *C. elegans* after infection with Mel^−3^ and comparison with the corresponding wild-type strain. (**a**) Survival of *C. elegans* showed a significant difference between Mel^−3^ and wild-type infection. n = 30–40 individuals per test were used, every test was repeated 3 times. (**b**) survival of *G. mellonella* showed no significant difference between both infections. n = 10 individuals per test were used, every test was repeated 3 times.

The black-yeast like fungi are also characterized by their ability to produce melanin^1^. In *E. dermatitidis* melanin is known to prevent the fungi being harmed by the hosts immune system^25^. Using the *C. elegans* model, we additionally investigated a melanin-deficient mutant of *E. dermatitidis* (Mel^−3^) and its corresponding wild-type strain to ensure that the death of nematodes was dependent on fungal virulence factors (Fig. 9). The killing quality of the wild-type strain (death rate of *C. elegans*, 55%) was similar to that of the other tested *E. dermatitidis* strains. However, the killing quality of the Mel^−3^ strain was lower (death rate of *C. elegans*, 33%). The tested mutant Mel^−3^ tended to be less virulent against *G. mellonella* than its corresponding wild-type strain, but this difference was not statistically significant (Fig. 9b).

### No correlation of genotypes and virulence

**Figure 10:**
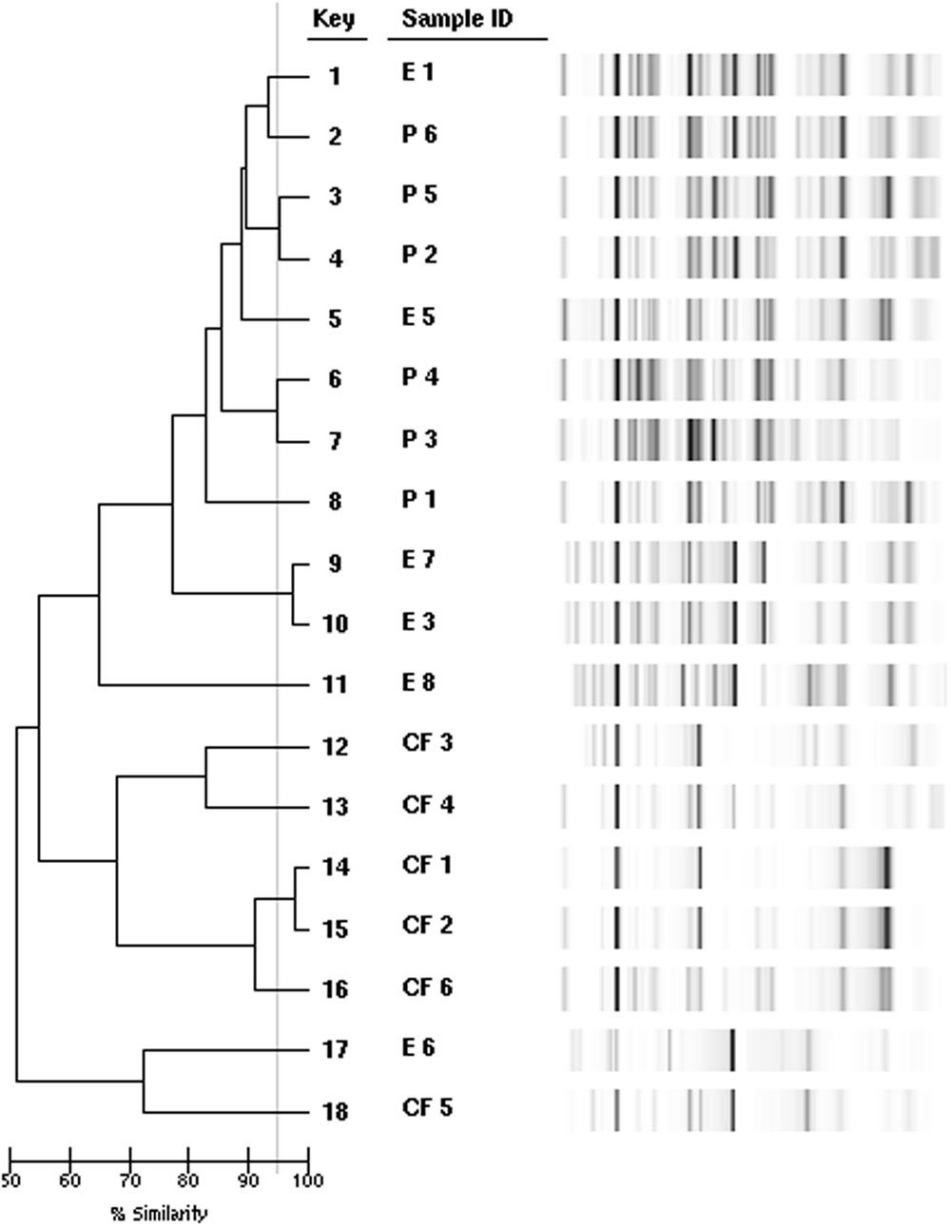
Dendrogram of cluster analysis estimated by DiversiLab®. 18 *E. dermatitidis* isolates of various origins were analysed and clustered after Kullback-Leibler algorithm. Line indicates 95% similarity.

In Fig. 10 the dendrogram of relatedness and gel images are depicted. The Asian patients and environmental strains showed a closer clonal relationship compared to the CF strains. No correlation between genotypes and virulence was present.

## Discussion

The virulence of the fungus *E. dermatitidis* has previous been studied primarily in murine models of infection. Intravenous infection with yeast-phase cells of *E. dermatitidis* confirmed the neurotropic character of this fungus and its preference for infecting host organs^8,26–29^. During infection, colony-forming unit (CFU) techniques^29^ and histologic analyses^8,26–28^ demonstrated exponential growth of *E. dermatitidis*. Infected organs exhibited micro-abscesses containing primarily yeast-phase cells. Interestingly, the observation of invasive hyphal structures was strain-dependent. Discussions of observed differences in the virulence of the tested *E. dermatitidis* strains are controversial; the differences depend on the production of melanin or on the observed strain-dependent production of invasive hyphal structures^8,28,29^. This is the first study to use alternative invertebrate infection models in combination, *C. elegans* and *G. mellonella*, as host organisms for determining the virulence of 18 wildtype *E. dermatitidis* strains.

All tested strains of *E. dermatitidis* exhibited pathogenicity against *C. elegans*. Differences in virulence were detected, but these differences were strain-dependent and were not associated with the defined strain groups. In addition we could not find any correlation between genotype and virulence. However, observations of *E. dermatitidis* morphology during infection with *C. elegans* indicated that CF strains can better maintain their location in the intestine of *C. elegans* than can strains isolated from systemic infections. The typical clinical picture of microbial infection in the nematode, including obstipation and death by cuticular burst, as described for *C. neoformans*, was also observed^18,20^. Strains from Asian patients (P) exhibited the development of hyphal structures during infection with *C. elegans*, but this development was highly dependent on the duration of infection. This finding, along with the fact that infected nematodes could remove P-strain cells from their intestine, indicates that strains from Asian patients (P) cannot retain their location in the intestine of *C. elegans* effectively enough to create a persistent infection.

This finding is similar to findings obtained from studies with *C. albicans* infection. This dimorphic yeast can produce lethal hyphal structures in *C. elegans* within the first 24 h after infection^30,31^. In contrast to *E. dermatitidis* cultures, *C. albicans* cultures grow rapidly, explaining the ability of the fungus to kill *C. elegans* within several hours by invasive hyphal formation^31,32^ combined also with the high affinity of this fungus for tissue cells^33^. Interestingly, biofilm experiments have shown that P strains of *E. dermatitidis* exhibit a higher potential for building biofilm than do CF strains or E strains^34^. Because the first step in biofilm formation is adherence to a surface structure, it is possible that the ability of *E. dermatitidis* to adhere to a surface depends on local environmental conditions and is also strongly strain-dependent.

As is the case with *C. elegans* infection, the invasive strains exhibited the highest rate of variability in virulence against *G. mellonella*. In comparison to the other strains, however, the P strains were significantly more virulent than either the CF strains or the E strains. Histologic analysis showed that some P strains also develop invasive hyphal structures more rapidly (within 24 h after infection) than do E strains or CF strains. Furthermore, histologic analysis found no hyphal structures in CF strains during the first 48 h after infection. Interestingly, histologic analysis also showed that strain P6 produced no invasive hyphae and exhibited relativly low virulence against *G. mellonella* caterpillars. These findings indicate an association between the development of invasive hyphal structures in *G. mellonella* during infection and the virulence of the *E. dermatitidis* strain. Our results are in accordance with a recently published work showing that the time-to-death of *G. mellonella* larvae was shorter for clinical *E. dermatitidis* isolates than environmental one^35^.

Previous studies have shown that the filamentous fungus *Aspergillus fumigatus* and the dimorphic yeast *Candida albicans* produce invasive hyphae during *G. mellonella* infection. However, the association between hyphal growth and virulence was demonstrated with hyphal-deficient mutants, and these studies showed less virulence not only depending on the loss of hyphal growth^36,37^. The decrease of growth kinetics in P-strain infections could be also explained by the increase in the number of hyphal structures after 72 h of infection. Growth kinetics during infection were determined by counts of CFUs; therefore, hyphal strucures could not be efficiently detected.

We used both infection models (*C. elgans* and *G. mellonella*) to investigate a melanin-deficient mutant (Mel^−3^) and its wild-type counterpart. The Mel^−3^ mutant is less resistant to an oxidative burst from the mammalian immune system, and its death rate during infection experiments is lower than during infection experiments using mice^25,28,38^. Both Mel^−3^ and its wild-type counterpart were pathogenic against *C. elegans* and *G. mellonella*. In *C. elegans*, the virulence of the wild-type strain was similar to that of *C. neoformans*, whereas the virulence of Mel^−3^ was lower in intensity, a finding indicating that 1,8-DHN melanin may play a role in the virulence of *E. dermatitidis* in *C. elegans*. Interestingly, no significant difference in the virulence of the two strains was found in *G. mellonella*. This finding that melanised fungi are less virulent against *G. mellonella* than are non-melanised mutants had been previously described for *Aspergillus fumigatus* and *C. neoformans*^39,40^, Melanin-deficient mutants of *A. fumigatus* exhibited higher virulence than did their wild-type counterparts. Jackson and colleagues speculated that the loss of melanin improves the interaction between the caterpillars’ immune system and the surface of fungal cells, followed by a stronger immune response that leads to self-harm of the insect by the toxic side products of the caterpillars’ own melanin synthesis^39^. On the other hand, non-melanisation could also lead to a larger release of toxic compounds from the fungal cell. The results of histologic analyses of the *E. dermatitidis* wild-type strains indicate that an overreaction of the caterpillars’ immune response is possible. The process of capsulation also continues 72 h after infection and creates thick layers of cells; however, the fungal cells inside are completely covered (Fig. 8c). Taken together, studies comparing melanization and virulence in *G. mellonella* might be limited as the results of the melanin-deficient mutant in the insect wax model are contrary to the results of *C. elegans* experiments and previous studies which used mouse models^8,38^

The caterpillar larva of the great wax moth *G. mellonella* is a common model host organism for infection experiments, to analyze the virulence of microorganism. Compared to *C. elegans* nematodes, the caterpillar insects are from higher complexity including the anatomy, development, and immune system. Whereas the immune response of *C. elegans* is limited in physical options, e.g. the cuticula barrier or various chemical compounds, the immune system of *G. mellonella* includes also a humoral immune response and specific immune cells localised in the hemolymph, called hemocyts.

The present results of the infection with *E. dermatitidis* melanin-deficient mutants and their wild types in *C. elegans* go along with those shown in mice infection experiments^26,28,38^. In both infection models, *C. elegans* and mice, melanin-deficient mutants were detected to express a decreased virulence and melanin was thus identified to contribute to the fungus virulence.

The *G. mellonella* model was so far only described for *E. dermatitidis* very recently in one publication^35^. The authors aimed to find differences between *E. spinfera* and *E. dermatitidis*. Compared to the published study we performed a more detailed pathogenicity analyses by using various clinical and environmental *E. dermatitidis* strains for the infection experiments.

In summary, the present study used two alternative invertebrate host model systems (*C. elgans* and *G. mellonella*) to study the virulence and putative virulence factors of the black yeast-like fungus *E. dermatitidis*. Unlike previous virulence studies, which used murine host organisms, this study used alternative host organisms that facilitate the parallel study of more than a few strains of interest. Fungal growth proliferates exponentially in murine and alternative hosts. Our observation of morphological development in *G. mellonella* during infection supports the hypothesis that the production of invasive hyphae increases the virulence rate. On the other hand, we observed the influence of melanin during virulence in *C. elegans* but not in *G. mellonella*. At the end, both alternative organisms have advantages and disadvantages for use as an alternative host for studying the virulence and the virulence factors of *E. dermatitidis* and should be evaluated by future studies.

## Material and Methods

### Strains and cultures

The strains used in this study, with their corresponding sources and areas of origin, are summarized in Table 2. The strains were identified by sequencing of the ITS regions. We isolated genomic DNA by using *E. dermatitidis* cells from 5 ml of a YPG culture that had been incubated for 48 h at 35 °C and shaken at 150 rpm. We adapted the glass bead method and the phenol/chloroform preparation reported by Hoffman^1^. Species from the clinical isolates were identified by sequencing the internal transcribed spacer (ITS)1 and ITS2 regions, as previously described^41^. The amplicons were sequenced by LGC Genomics (Berlin, Germany). Sequences were compared with reference sequences of *E. dermatitidis* by using the basic local alignment search tool (BLAST) developed at the National Center for Biotechnology Information (NCBI).

The study did not include patient’s details and did not result in additional constraints for the patients. All data were anonymously analyzed without patients’ consent due to the retrospective nature of the study. All analyses were carried out in accordance with approved guidelines.

Fungal cultures were maintained on malt agar (malt-extract agar plates; Oxoid Microbiology Products, Thermo Fisher Scientific, Carlsbad, CA, USA), and bacterial strains were maintained on blood agar with 5% sheep blood (Columbia agar plates; Oxoid). *C. elegans* strain N2 was maintained at 25 °C on nematode growth medium (NGM) agar overgrown with *E. coli* OP50. Nematode populations were bred and handled according to established procedures^30,42,43^. *E. coli* OP50 cultures were prepared for use as a food source for the *C. elegans* nematodes by incubation in 5 mL of Luria-Bertani (LB) liquid medium (10 g/L tryptone, 5 g/L yeast extract, and 171 mM NaCl) with shaking at 160 rpm for 24 h at 35 °C. As shown by several previous studies, *C. elegans* dies more rapidly after feeding on *E. coli* OP50 cultures in other media, such as brain heart infusion (BHI)^44^. After *E. coli* OP50 had been plated on NGM, the plates were incubated for 48 h at 35 °C.

Cultures of *E. dermatitidis* in yeast phase were obtained after incubation in 5 ml Sabouraud broth (SAB) (10 g/L peptone, 20 g/L D-dextrose at pH 5.5 with phosphoric acid) for 48 h. The liquid phase of the culture was then used to initiate a second culture in yeast peptone dextrose (YPD) liquid medium (1% yeast extract, 2% peptone, 2% D-dextrose [% = w/v]). That culture was incubated for 48 h at 35 °C with shaking at 250 rpm^27^. Cells were used after they had reached the late exponential phase.

### *C. elegans* infection model

In total, 20 *E. dermatitidis* strains were used to infect *C. elegans*. A *C. neoformans* yeast was used as an infection control, and cultures of *E. coli* OP50 were used as a life span control^17^. All tests used NGM agar with 5-fluoro-2′-deoxyuridine (FUDR; concentration, 0.45 mM; Calbiochem, San Diego, CA, USA) to prevent the production of progeny; additionally, ampicillin was used (concentration, 0.0025 mg/ml; Ampicillin sodium salt; Sigma-Aldrich) to prevent the growth of *E. coli* OP50^45^.

The nematode’s normal food sources are microorganisms that are involved in the decomposition of biological material; therefore, *C. elegans* can be infected by feeding on lawns of selected microorganisms^46^. The *E. dermatitidis* and *C. neoformans* strains were cultured in yeast-phase form, as described above. An inoculum of 10^7^ cells per plate was plated on NGM agar for optimal infection (size of inoculum determined previously, data not shown). The *E. coli* OP50 culture was prepared in LB medium and incubated at 35 °C for 24 h with shaking. Next, 25 µL of this culture was plated on prepared NGM agar without ampicillin. Before being used to infect *C. elegans*, all prepared plates were incubated at 25 °C for 24 h.

After synchronisation and washing according to standard *C. elegans* protocols^45^, 30 to 40 *C. elegans* nematodes in the L4 or the young adult developmental stage were pipetted onto the prepared lawns of *E. dermatitidis*, *C. neoformans*, or *E. coli* OP50^43^. Plates with *C. elegans* cultures were incubated at 25 °C in the dark. Infected *C. elegans* populations were examined daily for 7 days; dead and living nematodes were counted. Successful infection was defined as the exhibition of abnormal behaviour. Nematodes were considered dead when they did not respond to touch with a hair loop.

### Observation of morphology of *E. dermatitidis* in *C. elegans* infection

Synchronised L4 to young adult *C. elegans* nematodes were infected by feeding on lawns of *E. dermatitidis* or *C. neoformans* cells for 24 h or 48 h, as described above. After the inoculation, the nematodes were washed from the culture plates with M9 buffer and were centrifuged at 500 × g for 5 min. After this first washing step, they were moved to an agar medium containing drinking water and 2% agar (% = w/v). The infected nematodes were allowed to crawl over the agar for at least 30 min for removal of fungal cells from the outer cuticula. Afterward, the infected worms were moved to a 96-microwell plate, containing a mix of M9 buffer and brain heart infusion (BHI) broth (modified from Breger *et al*.)^32^: M9 buffer, 79%; BHI broth, 20%; cholesterol, 10 µL (concentration, 5 mg/L in ethanol; Carl Roth, Karlsruhe, Germany), ampicillin, 100 µL (concentration, 0.0025 mg/mL; ampicillin sodium salt; Sigma-Aldrich). The plates were implemented with 2 *C. elegans* worms per microwell, using 12 microwells per *E. dermatitidis* strain infection, and were observed daily for 72 h. Morphologic development of *E. dermatitidis* inside the infected *C. elegans* worm was observed by imaging (EVOS all-in-one digital inverted microscope; AMG, Bothell, WA, USA) and was listed together with information about death or health. Every test was repeated three times.

### *Galleria mellonella*: maintaining, handling, and storage

Previously described protocols were used for maintaining, handling, and storing *G. mellonella*^47,48^; these protocols were optimised for the laboratory location of the Institute of Medical Microbiology in Essen, Germany. The caterpillars, primarily the final larval stage, were obtained from a pet shop (Zoo und Angelsport GmbH, Bochum, Germany), at which the caterpillars had been maintained at 10 °C for 1 to 3 weeks (depending on demand). The caterpillars were transported under refrigerated conditions. In the laboratory, the caterpillars were kept in the dark at 22 °C to 24 °C under controlled humidity. The food mix for the bacteria was based on the so-called Haydak medium^49^ (glycerol, 400 g; liquid honey, 500 g; yeast extract, 100 g; wheat flour, 250 g; powdered milk, 200 g; polenta, 400 g; wheat bran, 250 g; all products were purchased at a local supermarket). Conditions and the health and fitness of the caterpillars were checked daily.

### *Galleria mellonella* infection model

*G. mellonella* caterpillars were infected as follows: Fungal strains were cultured in yeast phase, as described above. The concentration of fungal cells was determined with a Neubauer improved counting chamber (Carl Roth). Fungal cells were harvested and washed with sterile PBS (NaCl, 137 mM; KCl, 2.7 mM; Na_2_HPO_4_, 10 mM; KH_2_PO_4_, 1.8 mM; pH 7.2 with HCl). A cell solution containing 10^8^ cells per mL was prepared in PBS for infection. The concentrations of fungal cell solutions were checked so that the CFUs could be controlled. The cell concentration of the inoculum was determined in advance by titration experiments using inoculum from 10^6^, 10^7^, 10^8^, or 10^9^ cells per mL PBS. At a concentration of 10^8^ cells per mL we could detect a broad range of virulence in the comparative infection experiments.

Before *G. mellonella* caterpillars were used for experiments, they were stored for at least 48 h under the conditions described above for ensuring their health and fitness. Health was assessed by body colour: a yellow-cream colour with no dark marks indicated health; dark marks indicated an old or an acute reaction of the immune system against an unknown factor. Fitness was determined by the ability of the caterpillars to hang on a vertical cloth. Individual caterpillars that fell from a vertical cloth within the first 5 seconds after the cloth had been picked up were not used for infection. Caterpillars were weighed and classified by weight into 3 groups: 200 (±50) mg; 300 (±50) mg; and 400 (±50) mg. The area of injection was disinfected with a sterile cotton swab saturated with Cutasept F (Bode Chemie GmbH, Hamburg, Germany). Caterpillars were infected by injections of fungal cell solution directly into the haemocoel, piercing one of the last prolegs. A volume of 10 µL per 300 mg larvae was injected with a syringe pump (SyringePumpPro model LA-100; Landgraph Laborsysteme HLL GmbH, Langerhagen, Germany) at an injection rate of 2 µL/sec. Non-infection control caterpillars were injected with the fungus *C. albicans* in sterile PBS. Non-injection control caterpillars were not injected at all before incubation.

Infected caterpillars were incubated at 37 °C and were observed daily for up to 14 d. Dead caterpillars were removed from incubation, and *E. dermatitidis* were recultured from the caterpillars’ tissue.

### Recultivation of *Exophiala dermatitidis* and determination of fungal burden kinetics

Fungal cells for recultivation were taken from dead infected *G. mellonella* caterpillars after incubation for 5 min in 70% ethanol and were then transferred into FastPrep lysis M tubes (MP Biomedicals, LLC, Santa Ana, CA, USA) containing 500 mL fungal yeast extract-peptone-dextrose (YPD) medium. The prepared tubes were inserted into the MagNa Lyser (Roche, Grenzach-Wyhlen, Germany) for mechanical lysis by automatic shaking for 15 sec at 7000 × g. Next, 10 µL of the lysed tissue was transferred to 300 µL PBS. An aliquot of 10 µL of this solution was plated on Sabouraud dextrose agar (Oxoid) and incubated at 35 °C for at least 48 h.

For analyses of burden kinetics of *E. dermatitidis* in *G. mellonella*, infected caterpillars were used 2 h, 6 h, 24 h, 48 h, or 72 h after infection. Caterpillars were lysed as described above. Lysis solutions were prepared at dilutions of 10^−1^, 10^−2^, or 10^−3^; at 72 h the lysis solution was also prepared at a dilution of 10^−4^. An aliquot of each dilution was plated on Sabouraud dextrose agar plates containing and were incubated for 48 or 72 h at 35 °C. CFUs were counted, and the burden per mg caterpillar tissue was evaluated. CFU experiments were performed in duplicate and were repeated independently.

### Histologic analysis of infected *G. mellonella* caterpillars

Infected caterpillars were removed from incubation 24 h, 48 h, or 72 h after infection and were prepared as previously described by Perdoni and colleagues^50^. Infected caterpillar individuals were selected randomly and were moved into a 1.5-mL reaction tube and then inoculated with 100 µL of 10% buffered formaldehyde solution (Histofix, Carl Roth) by injection directly into the haemocoel of caterpillars. The caterpillars were then covered completely with a formaldehyde solution. The prepared caterpillars were incubated at 4 °C for at least 48 h so that optimal fixation of inner and outer morphological structures could be ensured. Fixed caterpillars were cut into halves longitudinally and embedded in paraffin wax. Optimal contrast and visualisation of caterpillar organs were obtained with HE staining; optimal contrast of fungal cells was obtained by staining with Grocott silver. The prepared and stained slides were analyzed by light microscopy (AxioScope; Zeiss, Jena, Germany), and observations were saved by photography (AxioScope Photo System; Zeiss).

### Strain typing for the investigation of genetically relationship

For the investigation of the genetically relationship between the analysed *E. dermatitidis* strains, strain typing by repetitive sequenced-based PCR (DiversiLab®; bioMérieux, France) was carried out. For DNA isolation purposes, a modified RNA isolation kit (Maxwell® 16 tissue LEV total RNA purification kit, Promega Mannheim). DNA concentration was determined using the NanoDrop® 1000 spectrometer (PeqLab, Erlangen). In cases of DNA concentrations below 35 ng/µL, a DNA precipitation with isopropanol was used. A PCR was carried out and products were run on agilent bioanalyser (fungal kit), while the DiversiLab® software was used for retrospective analysis of DNA fragment sizes. Results were displayed in an electropherogram with according signal profiles. The algorithm after Kullback-Leibler was applied for following cluster analysis.

### Statistics

All infection experiments using *C. elegans* and *G. mellonella* were repeated independently in triplicate. During comparative virulence studies, experiments were performed at least in duplicate and were repeated independently. The analysis of comparative hyphal growth was repeated independently in triplicate.

The Prism6 statistical program (GraphPadPrism, version 6.03; GraphPad Software, Inc., La Jolla, CA, USA) was used for all statistical analyses. The level of statistical significance was set at *P* ≤ 0.05. For comparison of hyphal growth, *t*-tests were used to detect differences between the defined *E. dermatitidis* strains. Survival curves for *C. elegans* and *G. mellonella* infection were plotted with the Kaplan–Meier Estimator. For identifying parametric or nonparametric data in the survival assay, means and medians of median-survival were compared. Parametric data were identified by a discrepancy of 1.0 or lower. For calculating mean survival curves from duplicates or triplicates, or for showing differences in virulence between the defined strain groups, the raw data from all included experiments were summarised and calculated as one experiment in the data sheet. Differences in estimated survival rates were compared with the Mantel–Cox log-rank test.

## Data Availability

The datasets generated during and/or analysed during the current study are available from the corresponding author on reasonable request.
